# Latitudinal gradients and sex differences in morphology of the Black Oystercatcher (*Haematopus bachmani*)

**DOI:** 10.1002/ece3.70115

**Published:** 2024-09-14

**Authors:** Hannah Roodenrijs, Lena Ware, Cole Rankin, Mark Maftei, J. Mark Hipfner, Brian H. Robinson, Daniel Esler, Heather Coletti, David J. Green

**Affiliations:** ^1^ Centre for Wildlife Ecology, Department of Biological Sciences Simon Fraser University Burnaby British Columbia Canada; ^2^ Canadian Wildlife Service Northern Region Environment and Climate Change Canada Whitehorse Yukon Canada; ^3^ Raincoast Education Society Ucluelet British Columbia Canada; ^4^ Environment and Climate Change Canada Science and Technology Branch Delta British Columbia Canada; ^5^ Alaska Science Center, U.S. Geological Survey Anchorage Alaska USA; ^6^ Southwest Alaska I&M Network U.S. National Park Service Anchorage Alaska USA

**Keywords:** Allen's rule, Bergmann's rule, bill length, wing shape, Black Oystercatcher, reversed sexual dimorphism

## Abstract

Environment and behavior are widely understood to affect bird morphology, which can lead to differences among subspecies or populations within a wide‐ranging species. Several patterns of latitudinal gradients in morphology have been described, though Allen's and Bergmann's rules are the most well‐known and have been tested and confirmed across a diversity of taxa and species. These state that individuals at higher latitudes will have larger bodies (Bergmann's Rule) but smaller extremities (Allen's Rule) to conserve heat in colder climates. Migratory behavior also can influence avian morphology, particularly wing shape, where migratory birds tend to have longer, more pointed wings than residents. The Black Oystercatcher (*Haematopus bachmani*) is a large, partially migratory shorebird species restricted to intertidal habitats and distributed from Alaska to Baja California, spanning about 35° of latitude. A large proportion of Black Oystercatchers that breed in Alaska are migratory, where nearly all individuals breeding in British Columbia through the southern end of their range remain resident through the annual cycle. Their broad latitudinal range and diversity in migratory behavior may drive geographic variation in morphology. Here we evaluate three explanations for geographic variation in morphology of the Black Oystercatcher using data from seven sites across two regions: Alaska and British Columbia. We found evidence consistent with Allen's but not Bergmann's rule; birds in Alaska have shorter bills than those in British Columbia, and these findings held when controlling for body size using wing length. Despite regional differences in migratory behavior, we detected no difference in the wing shape of birds in Alaska and British Columbia. Differences between sexes and among sites suggest that multiple factors drive patterns of morphological variation in the Black Oystercatcher.

## Graphical abstract

This study tests whether populations of Black Oystercatchers (*Haematopus bachmani*) across Alaska, the United States, and British Columbia, Canada, follow patterns of latitudinal gradients in morphology. Specifically, we test for patterns of Allen's and Bergmann's Rules, as well as differences in wing morphology based on migratory strategy. This study provides support for Allen's Rule in the Black Oystercatcher; those in northern latitudes have shorter bills, though it does not find evidence of Bergmann's Rule or the influence of migratory strategy on wing shape in this species.

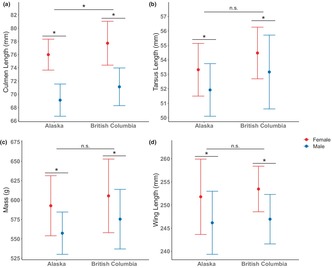

## 1 INTRODUCTION

Latitudinal variation in climatic and environmental conditions can impose selection pressures that drive latitudinal gradients in morphology, life history, and behavior (Bansal & Thaker, 2021; Cody, 1966; Díaz et al., 2013; James, 1970; Laurila et al., 2008). Latitudinal patterns of morphology have been described in a diverse array of taxa, including mammals (Alhajeri et al., 2020), birds (Symonds & Tattersall, 2010), and reptiles (Jaffe et al., 2016). Two of the earliest latitudinal patterns of morphology are referred to as Bergmann's Rule (Bergmann, 1847; Salewski & Watt, 2017) and Allen's Rule (Allen, 1877). Bergmann's rule refers to a general ecogeographical pattern that within a broadly distributed clade, populations and species found in colder environments and higher latitudes tend to consist of larger individuals, whereas populations and species with smaller individuals are found in warmer environments and at lower latitudes. Bergmann (1847) suggested that this pattern arises because larger‐bodied endotherms have a lower surface area to volume ratio and better heat retention than smaller‐bodied endotherms, making them better adapted to colder climates (Salewski & Watt, 2017). In reviews involving hundreds of species, most bird species followed expected patterns of Bergmann's rule in body mass and/or linear measurements (Ashton, 2002; Meiri & Dayan, 2003), though more recent meta‐analyses suggest that conformation to Bergmann's Rule may not be as extensive across species and taxa as previously thought (Henry et al., 2023; Riemer et al., 2018).

Allen's rule, an extension of Bergmann's rule, states that endothermic animals living in colder climates usually have shorter and rounder limbs, tails, and ears (in mammals) that allow them to retain more heat than closely related species in warmer climates (Allen, 1877). Consistent with this, in several avian families such as Spheniscidae (penguins), Laridae (gulls), and Sternidae (terns), bill length and bill surface area decrease with increasing latitude and decreasing minimum temperature across species (reviewed by Symonds & Tattersall, 2010). Allen's rule also applies to within‐species variation in bill morphology. For example, in Australia, Pied Oystercatchers (*Haematopus longirostris*) at higher latitudes tended to have shorter bills than those at lower latitudes (McQueen et al., 2022). Recent studies on birds have shown that the highly vascularized bill can aid in thermoregulation and quickly dissipate heat after physical exertion (Schraft et al., 2019; Tattersall et al., 2017). Schraft et al. (2019) found that tufted puffins can lose as much as 10%–18% of their excess body heat through their bills after energetically expensive flights. These widely observed latitudinal patterns of morphology can be useful in delineating individuals into subspecies or groups of different breeding origins when captured in a sympatric nonbreeding location (Delingat et al., 2011; Maggini et al., 2016; Ross & Bouzat, 2014).

Harsher winter climates at higher latitudes also can select for migratory life histories, and comparative studies show that the proportion of migrants and migration distance increases with latitude both across and within species (Murphy et al., 2017; Newton & Dale, 1996; Slud, 1976). Morphological adaptations for migration occur in a range of taxa (Chapman et al., 2015; Flockhart et al., 2017; Lockwood et al., 1998). In birds, longer, more pointed wings with greater convexity reduce drag and allow for more energetically efficient flight during migration (Lockwood et al., 1998). With more migratory individuals and longer migration distance at higher latitudes, wing shape can also follow a latitudinal gradient within and across species (Fiedler, 2005). Interspecific differences in wing length, pointedness, and convexity have been observed among species within clades that vary in their migration distance (Marchetti et al., 1995; Minias et al., 2015). Intra‐specific differences in wing shape can be pronounced in species with distinct sub‐populations (Egbert & Belthoff, 2003; Förschler & Bairlein, 2011) or where migration is a fixed rather than a facultative trait (Mulvihill & Chandler, 1991; Pérez‐Tris et al., 1999). Wing shape variation linked to migratory strategies consequently has been used to distinguish between migrants and residents when both are present in the same area (de la Hera et al., 2007; Pérez‐Tris et al., 1999).

**FIGURE 1:**
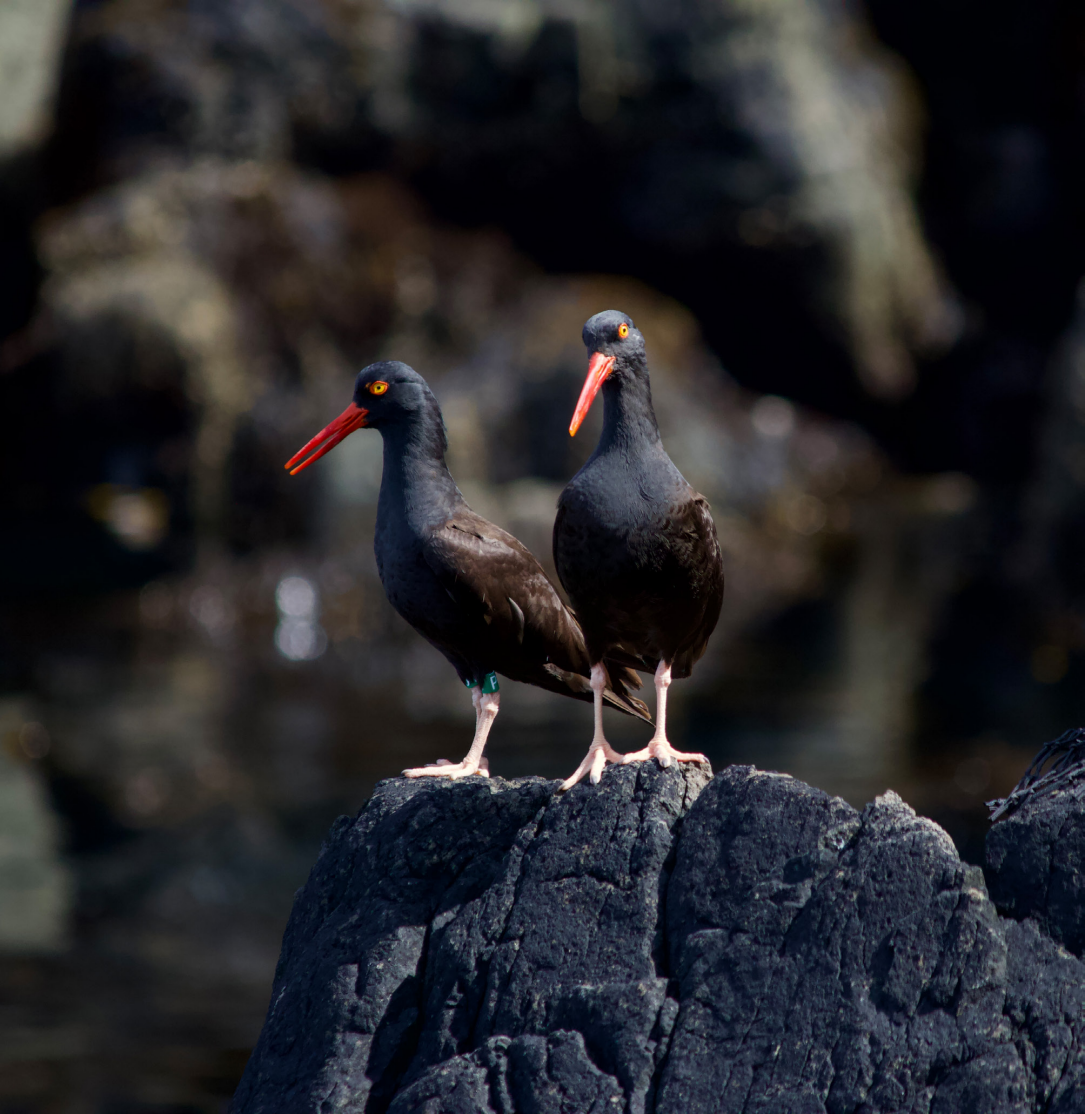
A pair of Black Oystercatchers (*Haematopus bachmani*) in Haida Gwaii, British Columbia.

The Black Oystercatcher (*Haematopus bachmani*) is a large, sexually dimorphic, and partially migratory shorebird found along the west coast of North America from the Aleutian Islands in Alaska to Baja California, Mexico (from latitudes of ca. 60° N to 26° N; Tessler et al., 2014; Figure 1). It is estimated that 80% of the global population is found in the northern portion of the range in Alaska and British Columbia (Tessler et al., 2014). Previous studies have documented geographical variation in bill morphology, wing length, and mass among breeding populations in Alaska (Guzzetti et al., 2008). The propensity to migrate varies with latitude: in Alaska, at least 50% of breeding individuals migrate south for the nonbreeding season (Johnson et al., 2010; Rankin, 2023), whereas birds breeding in British Columbia, Canada, are thought to be almost entirely resident (Johnson et al., 2010; Ware et al., 2023). In this study, we examine whether latitudinal variation in climate and migratory strategy are associated with differences in morphology of Black Oystercatchers in British Columbia and Alaska. Based on Allen's and Bergmann's rules, we predicted that birds captured in northern latitudes (Alaska) would have larger bodies with shorter legs and bills than those in British Columbia. Due to the higher proportion of migrants in Alaska, we predicted birds in Alaska would have more pointed and concave wings that improve migration efficiency.

## 2 MATERIALS AND METHODS

### 2.1 Study area

**FIGURE 2:**
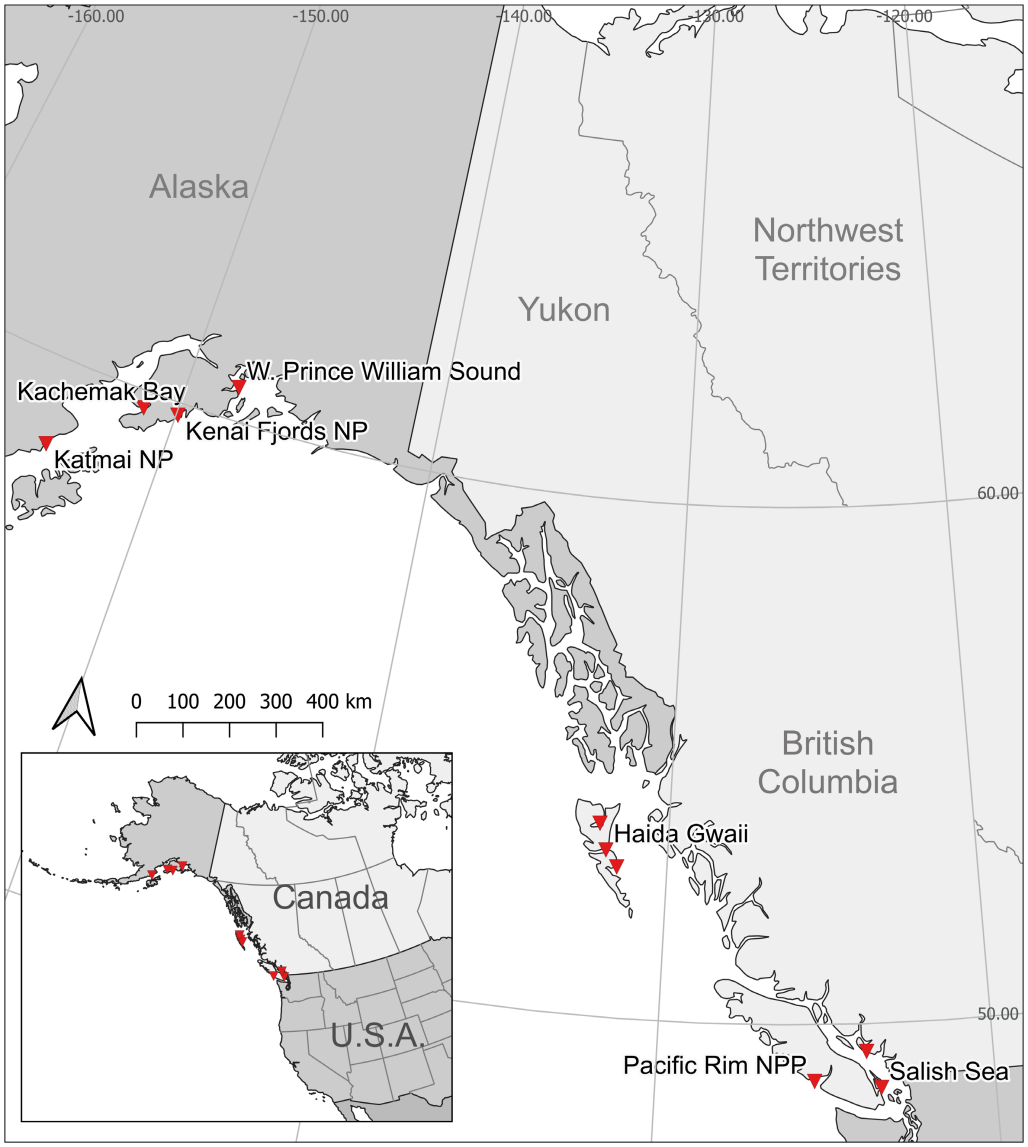
Study sites across Alaska, the United States, and British Columbia, Canada where Black Oystercatchers were captured between 2019 and 2022.

We studied Black Oystercatchers in British Columbia (BC), Canada, and Alaska, the United States of America (USA) (Figure 2). We captured birds at six locations in BC: Gulf Islands National Park and Preserve (48.77° N, 123.34° W), Pacific Rim National Park (48.94° N, 125.28° W), the Sunshine Coast Regional District (49.44° N, 123.65° W), Masset Inlet (53.63° N, 132.33° W), Skidegate Inlet (53.21° N, 132.11° W), and Laskeek Bay (52.91° N, 131.61° W) in Haida Gwaii. Due to their proximity and similarity in climate and topography, we combined data from the Gulf Islands and Sunshine Coast (hereafter referred to as the Salish Sea) and from Masset Inlet, Skidegate Inlet, and Laskeek Bay (hereafter referred to as Haida Gwaii). In Alaska, we captured birds at four sites: Katmai National Park and Preserve (58.23° N, 154.14° W), Kachemak Bay (59.61° N, 151.23°W), Kenai Fjords National Park (59.72° N, 149.70° W), and Western Prince William Sound (60.19° N, 147.91° W). We conducted fieldwork either in spring (March–April) or summer (June–August) in BC and in summer (May–July) in Alaska. All capture and handling of birds was conducted under permits provided by Simon Fraser University animal care and local and federal permits (United States banding permit number 20022, Canada banding permit number 10667Y, and Simon Fraser University animal use permit number 1218‐2021).

### 2.2 Field methods

We captured Black Oystercatchers using noose mats and noose lines with decoys (Mad River Decoys) and playback of calls (Foxpro Inferno, Lewiston, PA). All captures in Alaska targeted breeding and territorial birds during the summer season (May–July) in BC, captures targeted both territorial pairs (mainly during the May–August period) and nonterritorial groups (mainly during the February–March period). We banded each bird with a federal stainless‐steel band on the right tarsus and green plastic bands with a unique alpha‐numeric on each tibia (Haggie Engraving, Millington, MD). For each bird captured, we determined age based on plumage and the color of the bill and eye (Pyle, 2008). We assigned the sex of individuals by the extent of a black fleck in the iris of the eye, a method that is confirmed with molecular sexing to be 94% accurate (Guzzetti et al., 2008). For individuals with intermediate eye fleck scores, we used the sex of known mates along with culmen and tarsus lengths to infer sex.

**FIGURE A1:**
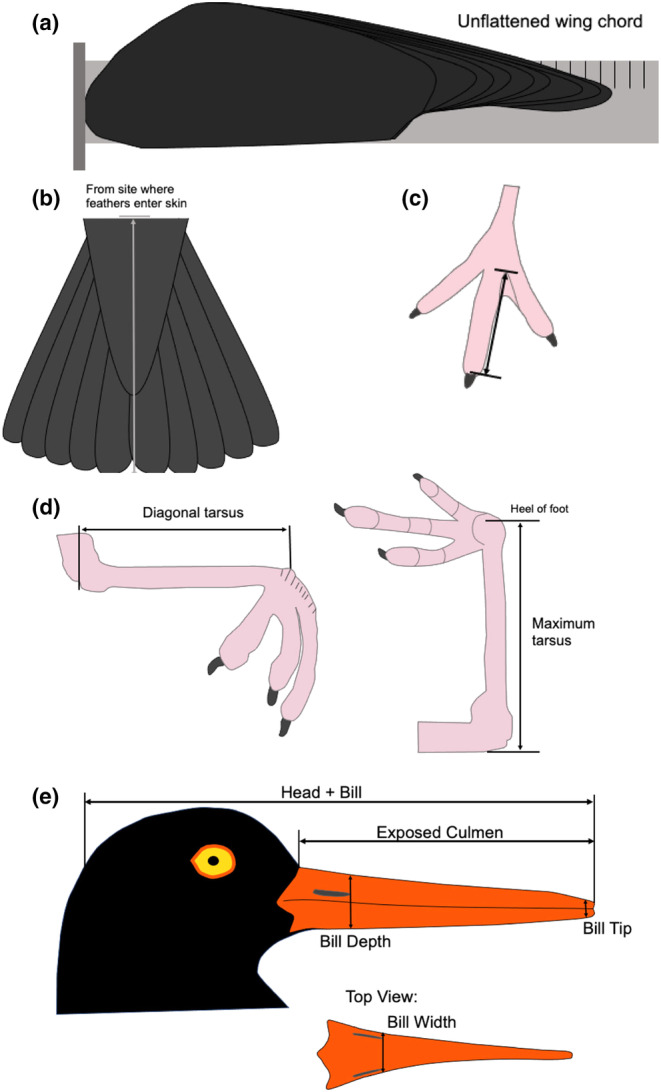
Schematic showing how morphological measurements were collected in the field: (a) wing cord, (b) tail length, (c) toe length, (d) two tarsus measurements, and (e) five bill measurements.

For each bird captured, we recorded ten morphological measurements (Roodenrijs et al., 2024; see Figure A1. for illustrations of each measurement). We weighed birds to the nearest 5 g using a spring balance (Pesola Medio 1000 g), and measured wing chord, tail length, and length of the middle toe to the nearest mm using a 1 mm‐unit ruler. We measured tarsus length using 0.1 mm‐unit calipers (SPI Polymid Dial 150 mm) in two ways: diagonal tarsus from the interstitial joint to the last leg scale before the toes (Pyle, 1997) and maximum tarsus length from the lower hind edge of the tibia to the heel of the foot. We described bill morphology with five measurements taken using 0.1 mm‐unit calipers (SPI Polymid Dial 150 mm): the length of the exposed culmen from the edge of the feathers to the tip, length of bill and head combined, bill depth at the nares, bill width at the nares, and depth of the bill at the tip.

### 2.3 Wing shape analysis

**FIGURE A2:**
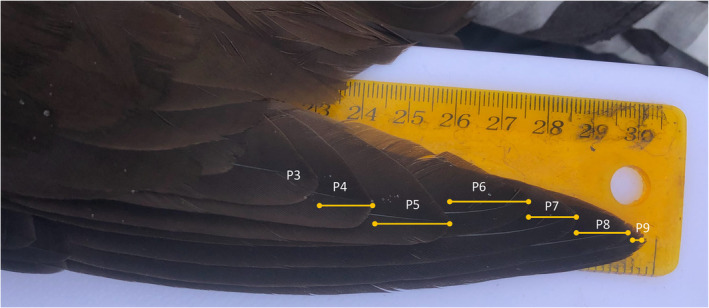
A diagram showing how the primary lengths were measured from photographs in Fiji. The yellow lines show the horizontal measurements made of the difference between each adjacent primary. These were subtracted from the wing chord length taken in the field (the length of the longest primary P9). The tenth primary (P10) is not visible in the photographs as it is shorter than the ninth primary (P9). *p < 0.05; **p < 0.01; ***p < 0.001.

Following Evered (1990), we calculated the length of each primary feather using the wing chord, the difference in length of the two outer primaries (P10 and P9), both measured in the field and the differences in length of each adjacent primary visible on photographs of the folded wing (see Figure A2) estimated using Fiji image processing software (Schindelin et al., 2012). Photographs were not taken in Haida Gwaii and the Sunshine Coast in 2019 because a structured photography protocol was not established. Depending on the extent of molt at the time of capture, we were able to measure the length of six to ten primaries per bird. We determined lengths of the nine outer primaries for 75 birds, and lengths of eight, seven, and six outer primaries for 100, 129, and 147 birds, respectively (Roodenrijs et al., 2024). The innermost primaries (P1 and P2) often were covered by the secondaries and rarely visible.

We quantified the wing shape of each bird using a size‐constrained components analysis (SCCA), as described by Lockwood et al. (1998), using R code (version 4.2.3, R Core Team, 2024) available from Stojanovic et al. (2020), which can be found in Roodenrijs et al. (2024). Size‐constrained components analysis uses lengths of primary feathers to calculate three measures of wing shape: the isometric size of the wing or overall wing size (C1), the pointedness of the wing (C2), and the convexity of the wing (C3). Initially, we quantified the wing shape of birds for which we had measurements of at least the nine outermost primary feathers measured (P10‐2). In this SCCA, the wing pointedness score was strongly influenced by loadings from the three outer primaries (P10, P9, and P8), whereas the wing convexity score was strongly influenced by the middle three primaries (P7, P6, and P5; Table A1). We also estimated wing shape for birds for which we had lengths of the 7 outer primaries (P10‐P4). In this SCCA, the pointedness and convexity scores were also heavily influenced by the length of the three outer primaries and the three middle primaries, respectively (Table A1). Pointedness and convexity scores obtained with the smaller and larger samples were correlated (pointedness‐C2, *r* > 0.95; convexity‐C3, *r* = 0.39). To maximize our sample size, we used wing shape estimates from the second SCCA in subsequent analyses.

### 2.4 Statistical analysis

**FIGURE A3:**
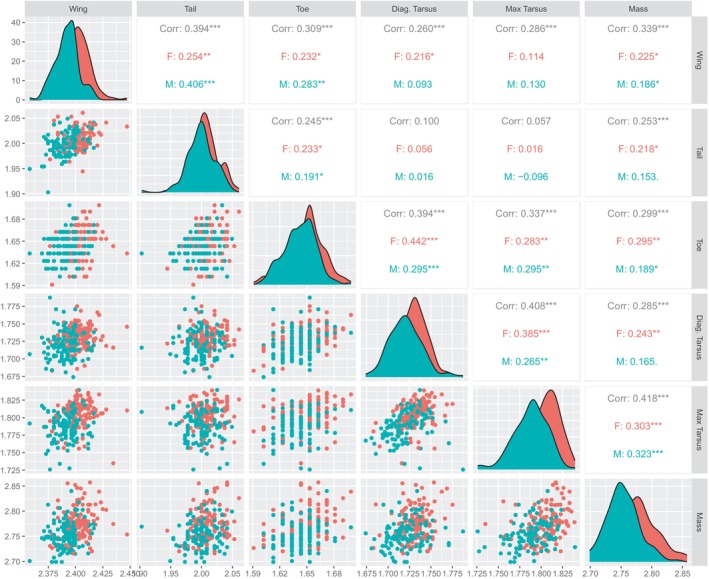
Correlation metrics of all body measurements of overall population (black text), female (in red), and male (in blue) Black Oystercatchers. The diagonal panels show distribution of measurements split by sex and the lower panels show the spread of the data. *p < 0.05; **p < 0.01; ***p < 0.001.

**FIGURE A4:**
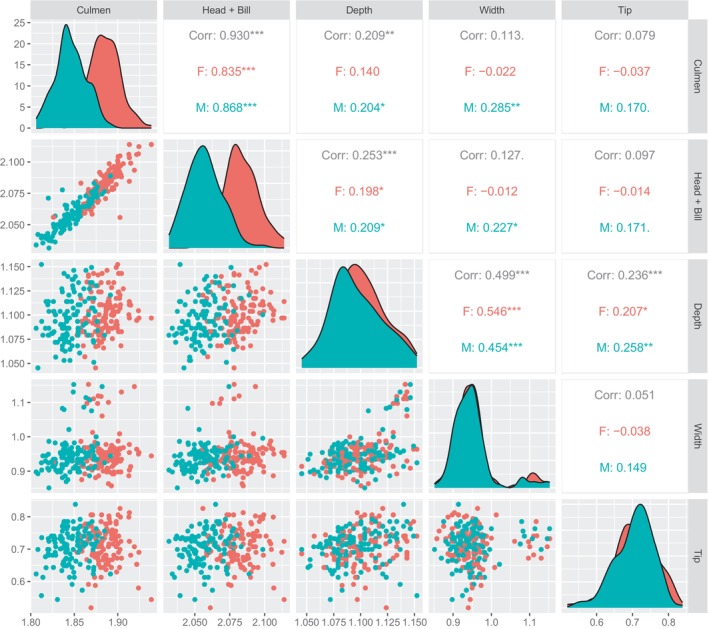
Correlation metrics of the five bill measurements of overall population (black text), female (in red), and male (in blue) Black Oystercatchers. The diagonal panels show distribution of measurements split by sex and the lower panels show the raw data. *p < 0.05; **p < 0.01; ***p < 0.001.

There was considerable collinearity in the morphological measures and mass (Figure A3). The two tarsus measures were correlated (*r* = 0.60), so we retained only one, diagonal tarsus length (hereafter tarsus length) in subsequent analyses. The five bill morphology measures were also correlated (culmen and head + bill, *r* = 0.93; bill depth and width, *r* = 0.50; bill depth and tip, *r* = 0.24), so we retained only exposed culmen length (hereafter referred to as culmen length), due to its wide use in avian morphology literature, and bill depth as measures of bill shape (Figure A4). In BC, birds caught in March were, on average, 24.5 g heavier than birds caught in June and July (*t* = −3.37, *p* = .001). We therefore adjusted the mass of birds to account for seasonal differences in mass prior to conducting the MANOVA (see below).

We evaluated Bergmann's and Allen's rule by comparing the morphology of birds captured in BC and Alaska using log‐transformed mass and six log‐transformed morphological measures. We decided to compare Alaska and BC in a two‐group analysis rather than running the analyses by latitude because we did not have a continuous sampling of latitudes, and the Alaska sites spanned a small range of latitude. Mass, wing length, and tail length provided indices of overall body size, metrics relevant to Bergmann's rule (James, 1970), while toe length, tarsus length, culmen length, and bill depth are linear measures of appendages whose length may influence heat retention and may be reflective of Allen's rule (Nudds & Oswald, 2007; Symonds & Tattersall, 2010).

We first evaluated whether there was any evidence for regional differences in morphology using a MANOVA that controlled for sex differences in morphology and seasonal differences in mass. We subsequently used linear mixed models to evaluate regional variation between Alaska and BC in each of the seven measurements while accounting for both sex differences and finer‐scale geographic variation associated with sampling sites within Alaska and BC. In these models, we used the individual morphological trait as the independent variable with sex, region, and an interaction between sex and region as fixed effects and site and bander as random terms. In the model for mass, we used the unadjusted, log‐transformed measurements, which included a random term of season to account for any seasonal differences in mass. We also conducted a post‐hoc size‐adjusted analysis of culmen length to confirm that sex and regional differences in culmen length did not arise due to differences in structural size by including wing length as an additional fixed effect in the culmen model. Finally, we used a series of ANOVAs to further explore site‐specific variation in the morphology of Black Oystercatchers in BC and Alaska, separately.

We next evaluated whether there was evidence for regional differences in wing shape of Black Oystercatchers. In these linear mixed models, we used wing pointedness (C2) and wing convexity (C3) scores as independent variables with sex, region, and an interaction between sex and region as fixed effects and site as a random term.

Finally, we used quadratic discriminant analyses (QDAs) to evaluate whether we could use morphological measurements and wing shape to distinguish between males or females captured in BC and Alaska. We chose to use a quadratic rather than linear discriminant analysis after Box's *M* test showed that the covariance matrices among the two groups were not equal (Box's *M* test for females: Chi‐Sq = 43.94, df = 28, *p* = .028; males: Chi‐Sq = 72.30, df = 28, *p* < .001). The analyses were run separately for each sex using the MASS package (Venables & Ripley, 2002). We randomly assigned 75% of the data to a “training” dataset to run the models and tested the model accuracy using the remaining 25% of the data in the “test” dataset. Sample sizes for the body morphometrics and wing shape differed, so initial QDAs included only the six log‐transformed body morphometrics and log‐transformed mass. Further LDAs added the pointedness and convexity scores to test if their addition improved model prediction accuracy.

## 3 RESULTS

We captured 251 adult Black Oystercatchers (Alaska *n* = 116, BC *n* = 135; Table 1) over 4 years (2019 *n* = 111; 2020 *n* = 28; 2021 *n* = 56; and 2022 *n* = 56; Table 1). Time spent in the field was limited in 2020 due to restrictions imposed during the COVID‐19 pandemic. Females (*n* = 125, 49.8%) and males (*n* = 126, 50.2%) were similarly represented in the dataset (Roodenrijs et al., 2024).

**TABLE 1:** Morphological variation in Black Oystercatcher from Alaska and British Columbia. We report means ± SD and range of raw data. Results of linear mixed models on log‐transformed data are reported below with statistically significant differences in bold. Random effects for both site and bander are reported as variance ± standard deviations.

	Wing		Tail		Toe		Tarsus		Culmen		Bill depth		Mass	
*Alaska*														
Male (*n* = 57)	247.1 ± 5.8 (235–261)		100.6 ± 5.4 (80–110)		43.8 ± 2.0 (40–50)		51.9 ± 1.8 (48–56.7)		69.1 ± 2.4 (64–76.2)		12.4 ± 0.6 (11.3–14.2)		557.3 ± 27 (500–597)	
Female (*n* = 59)	251.8 ± 8.1 (238–280)		103.0 ± 4.9 (88–113)		44.3 ± 2.1 (39–49)		53.3 ± 1.8 (48.2–56.9)		76.0 ± 2.3 (71.3–83)		12.6 ± 0.7 (11.1–14.2)		592.7 ± 39 (525–702)	
*British Columbia*														
Male (*n* = 69)	246.4 ± 4.9 (234–256)		99.5 ± 4.6 (90–112)		44.0 ± 1.6 (40–48)		53.2 ± 2.6 (47.2–61.3)		71.1 ± 2.8 (64.3–77.9)		12.6 ± 0.7 (11.1–14.1)		575.4 ± 38 (500–692)	
Female (*n* = 66)	253.5 ± 5.0 (245–268)		101.6 ± 4.0 (92–110)		45.0 ± 2.0 (40–50)		59.6 ± 1.8 (51.3–59.6)		77.7 ± 3.3 (67–86.8)		12.8 ± 0.6 (11.4–14.1)		605.4 ± 47 (520–720)	
*lmm results*														
Sex	***t* = −5.10**	***p* < .0001**	***t* = −3.07**	***p* = .002**	*t* = 1.63	*p* = .104	***t* = −3.77**	***p* < .001**	***t* = −13.51**	***p* < .0001**	*t* = −1.54	*p* = .125	***t* = −5.22**	***p* < .0001**
Region	*t* = 1.40	*p* = .184	*t* = 1.40	*p* = .184	*t* = 1.42	*p* = .17	*t* = 2.03	*p* = .065	***t* = 2.72**	***p* = .017**	*t* = 2.09	*p* = .051	*t* = 1.99	*p* = .065
Region*Sex	*t* = −0.31	*p*‐.761	*t* = 0.52	*p* = .603	*t* = −0.84	*p* = .404	*t* = −0.119	*p* = .905	*t* = 0.74	*p* = .462	*t* = −0.37	*p* = .713	*t* = 1.05	*p* = .295
*Random terms*														
Site	0.00001 ± 0.002		0		0		0.00002 ± 0.003		0.000005 ± 0.002		0.0000007 ± 0.001		0.000002 ± 0.001	
Bander	0.00002 ± 0.004		0.00006 ± 0.01		0.00006 ± 0.01		0.00002 ± 0.004		0		0.0002 ± 0.01		0.00006 ± 0.01	

**FIGURE 3:**
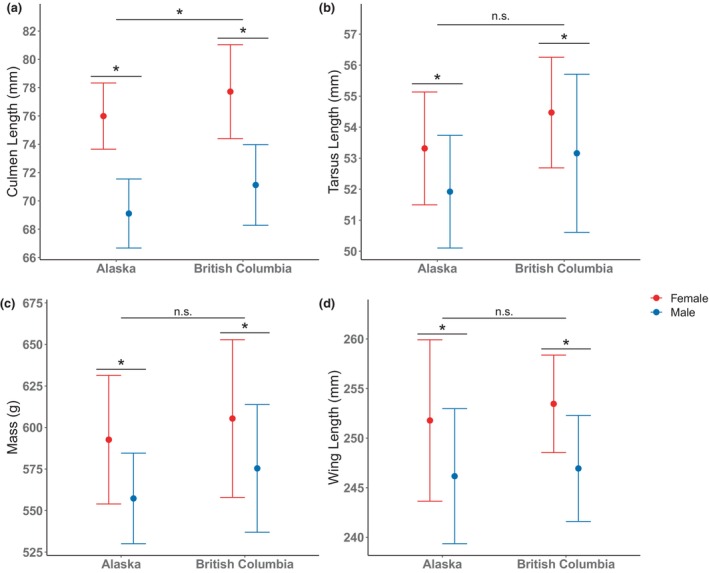
Means and standard deviations of selected morphological measurements by sex and region. Culmen (a) and tarsus (b) lengths represent appendage size to test for Allen's rule, while mass (c) and wing length (d) represent body size to test for Bergmann's rule. Asterisks above groupings represent statistically significant differences from the linear mixed models (those statistical analyses were performed using log‐transformed data).

We detected significant sex and regional variation in the morphology of Black Oystercatchers (MANOVA; sex: *F* = 58.24, *p* < .0001; region: *F* = 8.24, *p* < .0001). Females were generally larger than males in both Alaska and BC (Figure 3, Table 1). Females were heavier (by ca. 5%) and had longer wings, tails, tarsi, and culmen lengths than males (Table 1). Sex differences in culmen length (9%) were greater than sex differences in other morphological measures (2%–3%).

Black Oystercatchers in Alaska had shorter bills than those in BC (Figure 3, Table 1). This difference remained (*t* = 2.40, *p* = .03) when controlling for overall body size using wing length. Culmen lengths of females in BC were 2.5% longer than those in Alaska, while those of males in BC were 3.1% longer than those in Alaska. There was no evidence that birds in Alaska have larger bodies than those in BC: mass, wing length, and tail length did not vary regionally (Figure 3, Table 1).

We also detected finer‐scale variation in morphology of Black Oystercatchers (MANOVA; site: *F* = 3.06 *p* < .0001, sex: *F* = 58.24, *p* < .0001). This variation was primarily driven by morphological variation across sites in BC (Table A2). Wing length, tarsus length, culmen length, and bill depth varied across sites in BC, and only bill depth and mass varied across sites in Alaska (Table A2).

**FIGURE A5:**
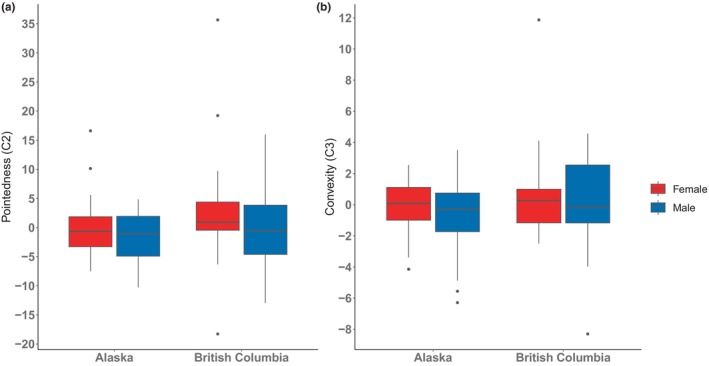
Sex and regional variation in wing shape of Black Oystercatchers, (a) wing pointedness (C2 scores) and (b) wing convexity (C3 scores). Higher pointedness scores indicate a more pointed wing (leading wing feathers longer compared to inner feathers) while higher convexity scores indicate longer primaries 4–5 compared to others around them.

We were able to quantify the wing shape (pointedness and convexity scores) of 137 birds. We found no regional or sex differences in wing pointedness (Figure A5; C2 score: region: estimate, BC = −2.76 ± 1.84, *t* = −1.50, *p* = .16; sex = estimate, *M* = −1.25 ± 1.39, *t* = 0.90, *p* = .37, region*sex: estimate = 1.46 ± 2.10, *t* = 0.70, *p* = .49) or wing convexity (C3 score; region: estimate, BC = 0.49 ± 0.64, *t* = 0.78, *p* = .45; sex = estimate, *M* = −1.52 ± 0.55, *t* = −0.95, *p* = .34, region*sex: estimate, BC*M = −0.30 ± 0.83, *t* = 0.36, *p* = .71).

The discriminant analysis confirmed that the six morphological traits differed between regions (females: *n* = 114, Wilks Lamba = 0.75, *p* < .0001; males: *n* = 117, Wilks Lambda = 0.79, *p* < .001). However, the QDA had limited ability to assign individuals to the correct region when using the six morphological traits and mass (females 62%, males 63%). The addition of wing shape metrics reduced the sample size in the QDA by about half but slightly improved the accuracy of assigning the region to individuals (67% for females and 81% for males).

## 4 DISCUSSION

Avian morphology can be shaped by climate (Allen, 1877; Salewski & Watt, 2017) and migratory strategy (Lockwood et al., 1998), which can vary with latitude. We present evidence that geographical variation in the morphology of Black Oystercatchers is consistent with Allen's rule, with Black Oystercatchers in Alaska having considerably shorter bills than those in BC. However, we found little evidence that geographical variation in morphology follows patterns consistent with Bergmann's rule or reflects regional differences in migration strategy.

Bergmann's rule predicts that body sizes of homeothermic individuals at higher latitudes will be larger than those at lower latitudes (Salewski & Watt, 2017), however, we found no evidence that Black Oystercatchers follow the predicted pattern. Nonmigratory species are more likely to comply with Bergmann's Rule than migrants, likely because they need to adapt to annual conditions in one location while migrants can track warmer temperatures by relocating for the nonbreeding period (McQueen et al., 2022; Meiri & Dayan, 2003). In our study, the presence of both resident and migrant individuals in the Alaska (Rankin, 2023) sample may have made it more difficult to detect patterns consistent with Bergmann's rule. Differences in body size by latitude could be more subtle in this species, and we may not have detected the pattern by only sampling the northern portion of the species' latitudinal range (Baldwin et al., 2023). Alternatively, pressure to conform to Bergman's Rule may be relaxed in species that conform to Allen's rule by reducing heat loss through altering appendage size, as the Black Oystercatchers in this study appear to do (Baldwin et al., 2023).

Allen's rule predicts that individuals at higher latitudes will have shorter extremities. The bill length and tarsus length of many bird species follow this pattern, though more species and families conform in bill length than in tarsus length (Symonds & Tattersall, 2010). Bills can evolve to aid in thermoregulation, and individuals can dissipate excess metabolic heat after physical exertion through convective heat loss from these featherless extremities (Schraft et al., 2019; Tattersall et al., 2017). We found that Black Oystercatchers in Alaska have shorter culmens than those in BC but not shorter tarsi. This finding of shorter culmen lengths in Alaska held when we ran an analysis that adjusted for body size. A recent study found that birds appear to be better able to regulate their blood flow and therefore heat loss through their tarsi than in the bill, explaining why perhaps more species, including Black Oystercatchers, conform to Allen's rule in the bill rather than in tarsus length (McQueen et al., 2023). Black Oystercatchers in Alaska experience colder average temperatures than those in BC, even in the breeding season, with the mean annual temperature of 11.2°C (min. 7.5°C, max 14.8°C) in June in Seward, Alaska (averages 2006–2020, NOAA National Centers for Environmental Information) versus a daily average of 15.2°C (min. 10.1°C, max 20.2°C) in June in Victoria, BC (1991 to 2020 Canadian Climate Normals Data, Environment and Climate Change Canada). Reduced bill size in Alaska may aid individuals in conserving heat by reducing convective heat loss through their bills. Behavioral thermoregulation follows a latitudinal gradient across many avian taxa, including shorebirds, with increased use of bill tucking at higher latitudes, providing further evidence that the heat loss through these appendages can make a difference in body temperature regulation in colder climates (Pavlovic et al., 2019). Alternatively, the differences could be driven by annual maximum temperatures, driving individuals in BC to have longer bills to increase heat dissipation in the warmer summer temperatures (McQueen et al., 2022; Youngflesh et al., 2022). Here, we show that sex differences in culmen length were much larger than regional differences, about 9% compared to 3%. This large difference in culmen length between sexes could arise as an allometric response to the larger body size of females or have evolved to allow for resource partitioning between the sexes (Nebel & Thompson, 2011; Selander, 1972). Given that Black Oystercatchers conform to Allen's Rule when only sampling the northern portion of the range, this suggests that this pattern holds strongly in this species.

Wing shape is argued to vary with migration strategy, and the selective pressure of migration often leads to individuals with longer and more pointed wings (Lockwood et al., 1998). With a higher proportion of migratory individuals and longer‐distance migrants at higher latitudes, wing shape variation may follow a latitudinal gradient (Fiedler, 2005; Somveille et al., 2013). We found no evidence of differences in the wing shape of Black Oystercatchers in Alaska and BC. Two factors may contribute to the absence of variation in wing shape in these populations. Regional variation in wing shape may be less likely when migration is a facultative trait or when migratory individuals travel relatively short distances (<4000 km, Förschler & Bairlein, 2011). Black Oystercatchers in Alaska are partial migrants, and it is unknown whether the decision to migrate is a fixed or facultative strategy (Johnson et al., 2010), but those that migrate (ca. 50%) travel only 800–1600 km (Rankin, 2023), which may be insufficient for wing shape effects on migration efficiency to have fitness benefits resulting in geographic variation in wing shape. Alternatively, adaptations to reduce predation risk and facilitate territory defense may be a stronger selective pressure on wing shape than migration in Black Oystercatchers. Black Oystercatchers are vulnerable to predation by Peregrine Falcons (*Falco peregrinus*) and Bald Eagles (*Haliaeetus leucocephalus*) and spend considerable time in territory defense against conspecifics, American Crows (*Corvus brachyrhynchos*), and Glaucous‐winged Gulls (*Larus glaucescens*) (Tessler et al., 2007). The evasion of predators and pursuit of intruders and predators often involves complex aerial maneuvers (Andres & Falxa, 2020). Predator avoidance and pursuit of intruders may therefore select more for wings that aid in quick, explosive flight (i.e., shorter rounder wings; Swaddle & Lockwood, 1998) in Black Oystercatchers in both Alaska and BC.

Researchers have successfully used differences in morphology and wing shape to discriminate migrants from residents or individuals from different breeding origins when found in the same site (Maggini et al., 2016; Neto et al., 2013; Pérez‐Tris et al., 1999). Here, despite some difference in morphology between birds in BC and Alaska, the linear discriminant analysis had limited ability to assign birds to their origin in BC or Alaska. Although assignment to region using the six morphological traits and mass was better than what would be expected by chance, the accuracy of our analysis was lower than other published studies using morphometric data in discriminant analysis (62%–81% in our study compared to about 85%–100% in other studies of songbirds; de la Hera et al., 2007; Delingat et al., 2011; Maggini et al., 2016; Neto et al., 2013). Limits in the ability to distinguish between birds from the two regions may be due to the considerable variation in morphology both within and across sites (this study, Guzzetti et al., 2008). Though we did not test for the covariation of morphology among sites with variables such as wave exposure, microclimates, and habitat types, there appear to be other factors that influence morphology on a local scale, particularly in BC. Intertidal invertebrate abundance and diversity can be highly variable (Zacharias & Roff, 2001), and the morphological differences among sites, particularly in bill shape, could indicate adaptation to local prey availability or the depth at which subsurface prey is buried based on local climate (Mathot et al., 2007). Nevertheless, the presence of sex, regional, and site differences in morphology suggests that multiple factors shape the morphology of Black Oystercatchers.

Black Oystercatcher populations show both regional and local variation in morphology (this study, Guzzetti et al., 2008). Regional variation in bill morphology is consistent with behavioral and physiological studies that suggest bill morphology plays a role in thermoregulation (Pavlovic et al., 2019; Tattersall et al., 2017). Further, our findings and those of Guzzetti et al. (2008) suggest that Black Oystercatchers may have other local adaptations to their environment that are not captured by Allen's and Bergmann's rules. Future studies including morphological measurements from individuals or museum specimens in the southern portion of the range could further our understanding of contemporary morphological variation in this species and possibly historical changes that have occurred. Finally, examining fitness consequences of variation in morphological traits that vary regionally or locally within Alaska and BC would further our understanding of how local climate, exposure, and prey availability influence the morphology of Black Oystercatchers.

## AUTHOR CONTRIBUTIONS

**Brian H. Robinson:** Investigation (equal); writing – review and editing (equal). **Cole Rankin:** Investigation (equal); writing – review and editing (equal). **Daniel Esler:** Conceptualization (equal); funding acquisition (equal); investigation (equal); writing – review and editing (equal). **David J. Green:** Conceptualization (equal); formal analysis (supporting); funding acquisition (equal); investigation (equal); methodology (equal); supervision (lead); writing – original draft (supporting); writing – review and editing (equal). **Heather Coletti:** Funding acquisition (equal); investigation (equal); writing – review and editing (equal). **Hannah Roodenrijs:** Conceptualization (equal); data curation (lead); formal analysis (lead); investigation (equal); methodology (equal); writing – original draft (lead); writing – review and editing (equal). **Lena Ware:** Investigation (equal); writing – review and editing (equal). **J. Mark Hipfner:** Funding acquisition (equal); writing – review and editing (equal). **Mark Maftei:** Investigation (equal); writing – review and editing (equal).

## CONFLICT OF INTEREST STATEMENT

The authors declare no competing interests.

## Data Availability

The data and code used to produce this article can be accessed at the USGS data repository under the title “Black Oystercatcher morphology and primary feather lengths in Alaska and British Columbia, 2019‐2022” 10.5066/P1KWBDRY.

## Appendix

**TABLE A1:** PCA loadings for each feather from the size‐constrained components analysis of wing shape using the 9 and 7 of the outermost primaries. Feather number P10‐P2 refers to the primary number starting from the leading edge of the wing, and counting inward with P10 brings the primary at the leading edge of the wing.

Feather	9 primaries included (*n* = 57)		7 primaries included (*n* = 94)	
	C2 pointedness	C3 convexity	C2 pointedness	C3 convexity
P10	−0.55433962	−0.12490243	−0.46298421	−0.3786398
P9	−0.42953453	−0.09447907	−0.37264311	−0.23417404
P8	−0.28333478	−0.07282673	−0.26151436	0.05508953
P7	−0.07136141	0.12722838	−0.07727983	0.46045562
P6	0.09367618	0.38861497	0.18390845	0.40834431
P5	0.17125323	0.33578135	0.34027198	0.27551386
P4	0.34135281	0.18879251	0.65024108	−0.58658948
P3	0.35947182	0.05871224		
P2	0.37281631	−0.80692122		

**TABLE A2:** Site‐specific morphological traits of female and male Black Oystercatchers in Alaska and British Columbia. Data are presented as means ± SD of the raw data. ANOVA results are reported below each trait, with significant results in bold. The ANOVA for mass in British Columbia included an additional fixed effect of season of capture to account for seasonal changes in mass (season: *F* = 10.47, *p* = .002).

Alaska								
Sex	Site	Wing	Tail	Toe	Tarsus	Culmen	Bill depth	Mass
Female	Katmai (*n* = 16)	252.4 ± 10.6	105.7 ± 4.8	44.3 ± 2.0	53.5 ± 1.9	77.3 ± 2.0	12.7 ± 0.5	584.4 ± 30.7
	Kachemak Bay (*n* = 7)	252.4 ± 10.6	105.6 ± 4.4	46.8 ± 1.5	53.4 ± 1.7	76.5 ± 3.1	12.6 ± 0.8	580 ± 20.6
	Kenai Fjords (*n* = 23)	253.0 ± 7.6	103.2 ± 3.7	44.9 ± 2.3	52.2 ± 1.9	75.7 ± 2.6	12.8 ± 0.9	600.7 ± 40.2
	W Prince William Sound (*n* = 13)	248.2 ± 7.8	102.0 ± 4.2	44.7 ± 1.2	53.9 ± 1.3	75.6 ± 1.4	12.6 ± 0.9	598.8 ± 52.2
Male	Katmai (*n* = 21)	244.9 ± 5.1	102.4 ± 4.5	43.3 ± 2.5	52.0 ± 2.0	69.8 ± 3.2	12.6 ± 0.7	544.4 ± 24.3
	Kachemak Bay (*n* = 8)	247.8 ± 2.1	101.0 ± 5.5	44.7 ± 0.8	51.4 ± 2.4	70.1 ± 3.1	12.3 ± 0.7	554.0 ± 31.6
	Kenai Fjords (*n* = 17)	247.2 ± 6.2	102.5 ± 3.7	44.3 ± 2.0	51.9 ± 1.6	69.0 ± 1.7	12.5 ± 0.8	560.9 ± 24.9
	W Prince William Sound (*n* = 10)	250.2 ± 6.0	100.3 ± 4.3	44.3 ± 1.8	52.0 ± 1.7	69.7 ± 2.8	12.3 ± 0.7	570.7 ± 22.4
Anova	Site	***F* = 7.75, *p* = .0007**	*F* = 1.50, *p* = .22	*F* = 2.43, *p* = .07	*F* = 1.53, *p* = .20	*F* = 0.18, *p* = .91	*F* = 2.35, *p* = .08	*F* = 2.55, *p* = .06
	Sex	***F* = 58.4, *p* < .00001**	***F* = 5.26, *p* = .02**	*F* = 2.38, *p* = .13	***F* = 11.17, *p* = .001**	***F* = 205.8, *p* < .00001**	***F* = 6.19, *p* = .01**	***F* = 26.1, *p* < .00001**
	Sex*Site	*F* = 1.76, *p* = .16	*F* = 0.70, *p* = .55	*F* = 0.94, *p* = .42	*F* = 1.09, *p* = .36	*F* = 1.06, *p* = .37	*F* = 0.30, *p* = .82	*F* = 0.26, *p* = .86
*British Columbia*								
Female	Haida Gwaii (*n* = 28)	255.5 ± 6.8	101.8 ± 4.9	44.9 ± 2.3	54.3 ± 1.6	78.4 ± 3.2	12.4 ± 0.3	619.2 ± 48.7
	Pacific Rim (*n* = 22)	253.5 ± 3.6	101 ± 3.6	45.1 ± 1.6	54.6 ± 2.1	77.9 ± 2.4	13.0 ± 0.4	612.6 ± 38.5
	Salish Sea (*n* = 16)	251.8 ± 3.5	102.6 ± 5.3	45.5 ± 2.0	55.1 ± 1.5	76.9 ± 4.1	12.5 ± 0.6	608.1 ± 44.4
Male	Haida Gwaii (*n* = 29)	248.1 ± 4.6	100.1 ± 5.4	43.8 ± 1.9	51.5 ± 2.0	69.8 ± 3.2	12.1 ± 0.8	591.0 ± 45.8
	Pacific Rim (*n* = 22)	245.5 ± 5.8	97.8 ± 5.2	43.4 ± 1.3	53.2 ± 1.9	72.6 ± 2.8	12.5 ± 0.5	568.1 ± 21.5
	Salish Sea (*n* = 19)	245.4 ± 4.6	98.3 ± 4.0	43.8 ± 1.9	53.1 ± 1.5	70.8 ± 3.1	12.6 ± 0.4	584.9 ± 38.7
Anova	Site	***F* = 4.32, *p* = .01**	*F* = 0.53, *p* = .59	*F* = 0.01, *p* = .99	***F* = 4.80, *p* = .01**	***F* = 3.71, *p* = .028**	***F* = 5.89, *p* = .004**	***F* = 0.64, *p* = .53**
	Sex	***F* = 50.21, *p* < .0001**	***F* = 9.54, *p* = .002**	***F* = 16.43, *p* = .0001**	***F* = 33.52, *p* < .0001**	***F* = 104.5, *p* < .0001**	*F* = 3.20, *p* = .08	*F* = 12.54, *p* = .0006
	Sex*Site	*F* = 0.19, *p* = .83	*F* = 0.62, *p* = .54	*F* = 0.31, *p* = .73	*F* = 1.10, *p* = .33	*F* = 2.09, *p* = .13	*F* = 1.94, *p* = .15	*F* = 0.54, *p* = .59
